# N-acetylcysteine promotes doxycycline resistance in the bacterial pathogen *Edwardsiella tarda*

**DOI:** 10.1080/21505594.2024.2399983

**Published:** 2024-09-06

**Authors:** Juan Guo, Qingqiang Xu, Yilin Zhong, Yubin Su

**Affiliations:** Department of Cell Biology & Institute of Biomedicine, National Engineering Research Center of Genetic Medicine, MOE Key Laboratory of Tumor Molecular Biology, Guangdong Provincial Key Laboratory of Bioengineering Medicine, College of Life Science and Technology, Jinan University, Guangzhou, Guangdong, China

**Keywords:** *Edwardsiella tarda*, N-acetylcysteine, doxycycline, antibiotic resistance, reactive oxygen species, efflux pump

## Abstract

Bacterial resistance poses a significant threat to both human and animal health. N-acetylcysteine (NAC), which is used as an anti-inflammatory, has been shown to have distinct and contrasting impacts on bacterial resistance. However, the precise mechanism underlying the relationship between NAC and bacterial resistance remains unclear and requires further investigation. In this study, we study the effect of NAC on bacterial resistance and the underlying mechanisms. Specifically, we examine the effects of NAC on *Edwardsiella tarda* ATCC15947, a pathogen that exhibits resistance to many antibiotics. We find that NAC can promote resistance of *E. tarda* to many antibiotics, such as doxycycline, resulting in an increase in the bacterial survival rate. Through proteomic analysis, we demonstrate that NAC activates the amino acid metabolism pathway in *E. tarda*, leading to elevated intracellular glutathione (GSH) levels and reduced reactive oxygen species (ROS). Additionally, NAC reduces antibiotic influx while enhancing efflux, thus maintaining low intracellular antibiotic concentrations. We also propose that NAC promotes protein aggregation, thus contributing to antibiotic resistance. Our study describes the mechanism underlying *E. tarda* resistance to doxycycline and cautions against the indiscriminate use of metabolite adjuvants.

## Introduction

*Edwardsiella tarda* is a Gram-negative facultative anaerobic bacteria and an important member of the *Enterobacteriaceae* family [1,2], which can infect fish, reptiles and humans [3–5]. *E. tarda* has a significant impact on the aquaculture industry and human health. Humans are infected by contact with or consumption of improperly cooked aquatic animals, which can cause gastrointestinal disease, bloodborne infections, and wound infections [5,6] and aquatic animals infected by the disease can develop severe symptoms such as ascites, hernia, exophthalmia and internal organ damage [7–9]. Because of the indiscriminate use of antibiotics, drug-resistant strains of *E. tarda* have been found in many regions [10–12] what poses antibiotic resistance challenges. Therefore, how to fight antibiotic resistance is very important. A study shows that haemolysin activator (*eha*) gene has an impact on biofilm formation, adhesion, and pathogenicity of pathogenic strain ET13 [3]. *E. tarda* phage can be used as a potential bio-therapeutic candidate to control multi-drug resistant *E. tarda* [4]. Antimicrobial resistance also can be reduced through environmental management, control of host interactions, sanitation and hygiene, health monitoring, nutritional support, and applications and development of vaccines, probiotics and drugs [5]. Although developing new antibiotics remains a primary strategy, challenges such as marketing difficulties and disproportionate research and development costs persist [13]. A potential solution for treating bacterial resistance lies in combinatorial drug therapy, which reduces the pressure to develop new antibiotics [14,15].

Drug combinations are used to address bacterial resistance through various methods. For example, 1. combinations of antibiotics, such as cephalexin and gentamicin can effectively assist in eradication of *E. tarda* [16], 2. non-antibiotic combinations, such as carvacrol and cymene synergistically assist in eradication of *E. tarda* [17], 3. combinations of non-antibiotics and antibiotics improve antibiotic sensitivity [18]. Many non-antibiotic substances, such as glucose [19], aspartate [20], α-ketoglutarate [21], and fructose [22], assist in eradication of *E. tarda*. However, non-antibiotic substances promote antibiotic resistance in *E. tarda* by effecting glutathione synthesis [23], redox levels [24], and fatty acid synthesis [25]. The inappropriate use of non-antibiotics increases the risk of antibiotic resistance, emphasizing the need for careful selection and use of non-antibiotic substances to manage bacterial resistance.

N-acetylcysteine (NAC) is a drug with anti-inflammatory, antibacterial, and antioxidant activities [26–28]. This drug is found in natural sources and is available in the treatment of various diseases [29]. While studies suggest NAC can reduce antibiotic resistance in bacteria by eliminating antibiotic-promoted SOS response [30], increasing cell membrane permeability [31], and inhibiting biofilm formation [32], there is evidence that NAC protects bacteria from being killed when used in combination with carbonyl cyanide 3-chlorophenylhydrazone [33]. In a recent study, NAC blocked the death of methicillin-resistant *Staphylococcus aureus* induced by uracil and gentamicin [34]. A recent review of NAC included several studies that examined its impact on bacterial resistance but focused on the role of NAC in biofilm formation and reactive oxygen species (ROS), with limited information about its effect on minimum inhibitory concentrations (MICs) [35]. However, the mechanisms by which NAC affects antibiotic resistance in bacteria have not yet been fully explored. In this study, we show that NAC can promote resistance to many antibiotics, particularly those of the tetracycline class, and explore the mechanisms by which NAC protects bacteria from antibiotics. This study suggests that clinical use of NAC in combination with antibiotics should be considered with caution.

## Results

### NAC promotes resistance of *E. tarda* to doxycycline

**Figure 1.**
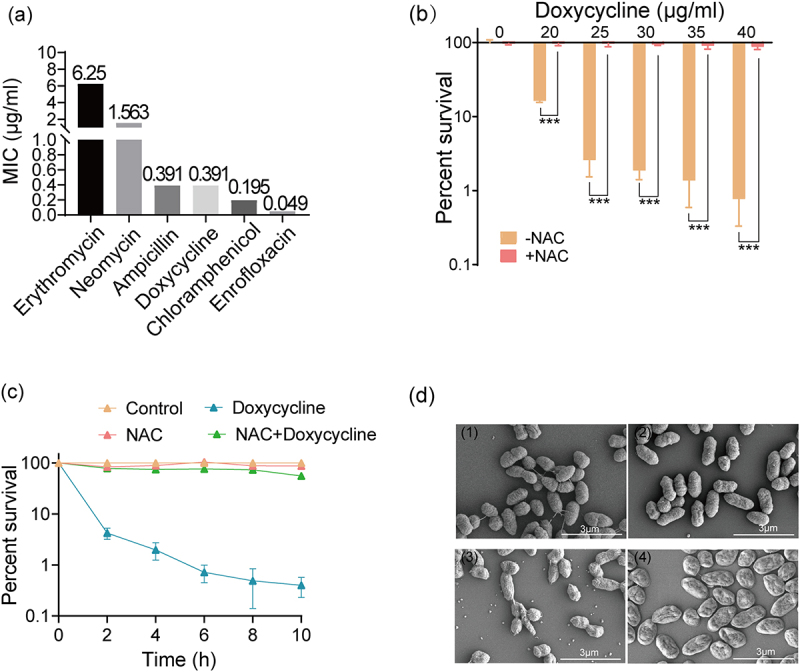
NAC affects doxycycline resistance. (a) MIC of different antibiotics: the x-axis represents antibiotics, and the y-axis corresponds to MIC (μg/ml). (b) Synergistic sterilization of different concentrations of doxycycline hydrochloride and 40 mM NAC: the y-axis corresponds to bacterial survival rate. (c) Time gradient sterilization experiment of 40 μg/ml doxycycline hydrochloride and 40 mM NAC. (d) SEM of ATCC15947: (1) control group, (2) NAC group-40 mM, 3) doxycycline group-40 μg/ml, (4) NAC+ doxycycline group. Data are presented as mean ± SEM (*n* = 3 biological replicates) and statistically significant differences are identified by t-test. ***p < 0.001.

The MIC of *E. tarda* ATCC15947 against various antibiotics was determined. According to the Clinical Laboratory Standards Institute (CLSI) standards, *Escherichia coli* ATCC 25,922 is a recommended reference strain for antibiotic susceptibility testing and ampicillin MIC range of ATCC25922 is 2–8 μg/ml. The result measured value was 3.125 μg/ml (Figure S1). Therefore, MICs of antibiotics were recorded using the same method for *E. tarda*. The MIC values of *E. tarda* ATCC15947 against various antibiotics (Figure 1a) were erythromycin (6.25 μg/ml), neomycin (1.563 μg/ml), ampicillin (0.391 μg/ml), doxycycline (0.391 μg/ml), chloramphenicol (0.195 μg/ml), and enrofloxacin (0.049 μg/ml). According to the CLSI standards, Enterobacteriaceae are considered sensitive if the doxycycline MIC ≤4 μg/ml. These results suggest that *E. tarda* ATCC15947 is sensitive to doxycycline. As NAC is a bactericidal substance [36], the impact of NAC alone on the survival rate of *E. tarda* was measured. After treatment with 50 mM NAC, the survival rate was significantly reduced to 68% but when *E. tarda* were treated with 40 mM NAC, the survival rate was 91% (Figure S2a). Therefore, 40 mM NAC was selected for the following experiments. Next, to determine whether there is synergistic effect of NAC and antibiotics, *E. tarda* were treated with 40 mM NAC in combination with concentration gradients of doxycycline, enrofloxacin, erythromycin, chloramphenicol, neomycin, and ampicillin. The bacterial survival rate was 1% after using doxycycline alone but increased to 87% when doxycycline and NAC were used in combination (Figure 1b). Similarly, the combination of enrofloxacin and NAC increased the survival rate from 1% to 42% (Figure S2b); erythromycin and NAC increased the survival rate from 5% to 90% (Figure S2c); chloramphenicol and NAC increased the survival rate from 15% to 90% (Figure S2d). However, the combined use of neomycin and NAC reduced the bacterial survival rate from 30% to 9% (Figure S2e) and NAC did not affect the susceptibility of *E. tarda* to ampicillin (Figure S2f). These results suggest that NAC promotes doxycycline, enrofloxacin, erythromycin, and chloramphenicol antibiotic resistance. In contrast, NAC synergizes with neomycin to kill bacteria and NAC has no synergistic effect with ampicillin.

Given that *E. tarda* ATCC15947 is sensitive to doxycycline, NAC combined with doxycycline had a significant effect on bacterial resistance. Therefore, we chose a combination of NAC and doxycycline for subsequent experiments. When treated with doxycycline alone, the bacterial survival rate decreased with time and then stabilized at 1% at 6 h; however, when NAC was used in combination with doxycycline, the survival rate remained at 50–80% (Figure 1c). In addition, when treated with doxycycline alone, the rod-shaped bacteria had a central depression but the combination of NAC and doxycycline reduced this depression (Figure 1d). Therefore, NAC alleviated the damage caused by doxycycline. NAC also synergized with other tetracycline antibiotics (minocycline or tigecycline) to induce resistance (Figures S3a and b). These data suggest that NAC induces resistance of *E. tarda* to tetracycline antibiotics.

### NAC activates pathways linked to the synthesis and metabolism of amino acids and the outer cell membrane

**Figure 2.**
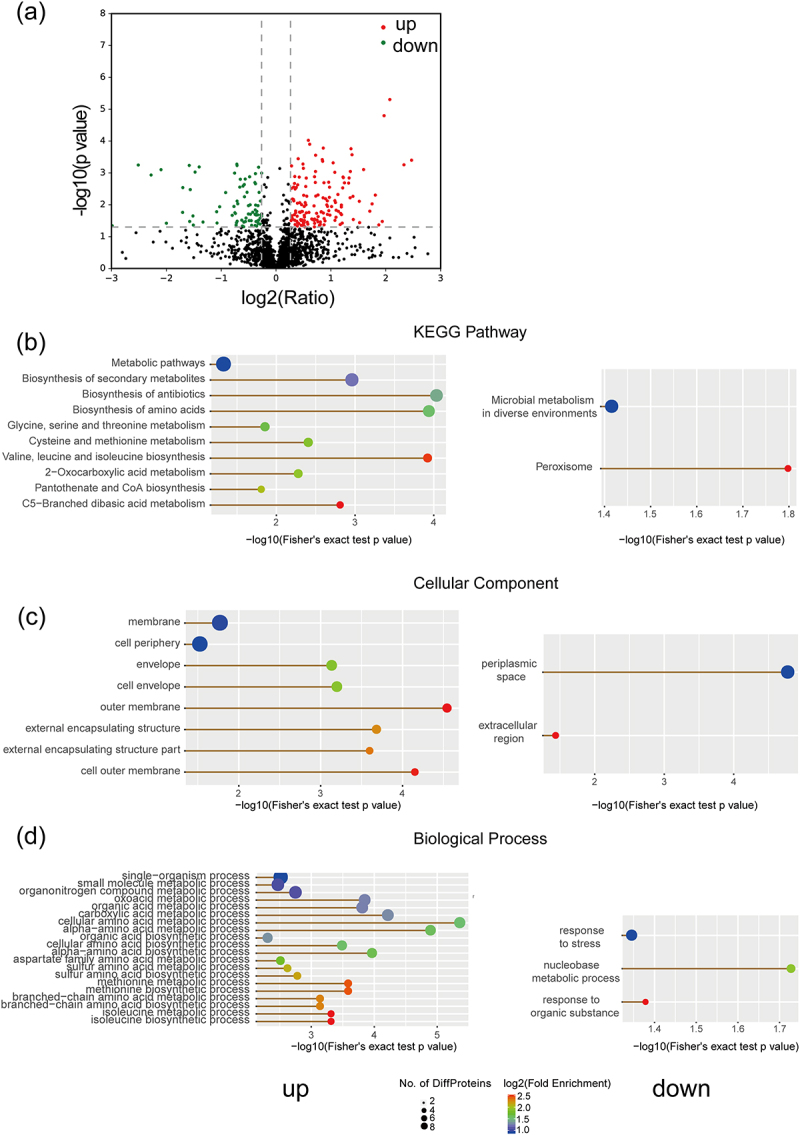
Proteomic analysis of untreated control and NAC groups. (a) Differential protein volcano plot: each point in the figure represents a specific protein, with red representing up-regulation and green representing down-regulation. (b) KEGG pathway of upregulated and downregulated proteins. (c) GO analysis (celluar component) of upregulated and downregulated proteins. (d) GO analysis (biological processes) of upregulated and downregulated proteins.

Proteomics was used for mechanistic analyses. The repeatability index of the untreated control and NAC group was high, indicating that the reproducibility of samples was good (Figure S4). Compared with the untreated control group, 232 proteins were differentially expressed in the NAC group; 151 proteins were upregulated and 81 downregulated after 40 mM NAC treatment (Figure 2a). The upregulated differentially expressed proteins (DEPs) were enriched in 10 Kyoto Encyclopedia of Genes and Genomes (KEGG) pathways, mainly involving amino acid synthesis and metabolism pathways. The downregulated DEPs were only enriched in two KEGG pathways (Figure 2b). In the Gene Ontology (GO) cellular component analysis, the upregulated DEPs were enriched for eight molecular functions (mostly involving the extracellular membrane) and downregulated DEPs were enriched in two molecular functions only (Figure 2c). In the GO biological process analysis, the upregulated DEPs were enriched in 20 biological processes, mostly involving the synthesis and metabolism of amino acids, and the downregulated DEPs were only enriched in three biological processes (Figure 2d). Therefore, based on proteomic data, we speculate that NAC treatment of bacteria upregulates amino acid synthesis and metabolism, as well as extracellular membrane-related pathways.

**Figure 3.**
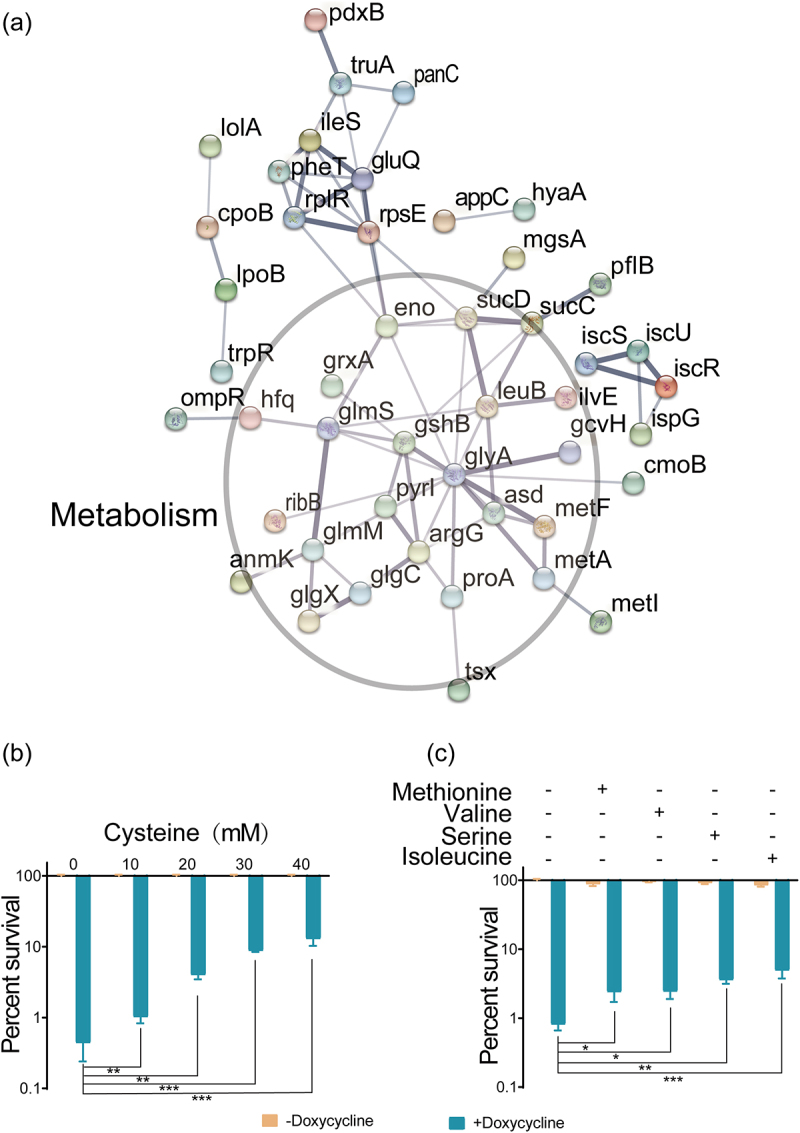
Antibacterial efficacy of doxycycline hydrochloride in the presence of various amino acids. (a) Predicted protein – protein interaction network. (b) Synergistic effects of different concentrations of cysteine and 40 μg/ml doxycycline on the survival rate of ATCC15947. (c) The synergistic effect of 40 mM amino acids (methionine, serine, isoleucine and valine) and 40 μg/ml doxycycline on the survival rate of ATCC15947. Data are presented as mean ± SEM (*n* = 3 biological replicates) and statistically significant differences are identified by t-test. *p < 0.05.**p < 0.01. ***p < 0.001.

To further understand the intracellular changes caused by NAC, STRING (Search Tool for the Retrieval of Interacting Genes/Proteins) analysis software was used and the analysis again showed that most of the upregulated DEPs were involved in amino acid synthesis and metabolism (Figure 3a). Next, we explored the impact of amino acids on the lethality of doxycycline. Cysteine is a direct conversion product of NAC and the bacterial survival rate was 1% after treatment with doxycycline alone but increased to 12% when doxycycline was combined with cysteine (Figure 3b). NAC increased the expression of enzymes related to amino acid synthesis (Table S2), such as the first enzyme involved in the methionine synthesis pathway (MetAS), serine hydroxymethyltransferase (GlyA), and isoleucine and valine synthesis related protein (IlvE). Therefore, in addition to cysteine, the effects of methionine, serine, isoleucine, and valine on the lethality of doxycycline were analysed. The bacterial survival rate was significantly increased when doxycycline was combined with methionine, serine, isoleucine, or valine (Figure 3c). These results suggest that the addition of NAC-associated amino acids promotes the doxycycline resistance of *E. tarda* ATCC15947.

### NAC reduces ROS levels in *E. tarda*

**Figure 4.**
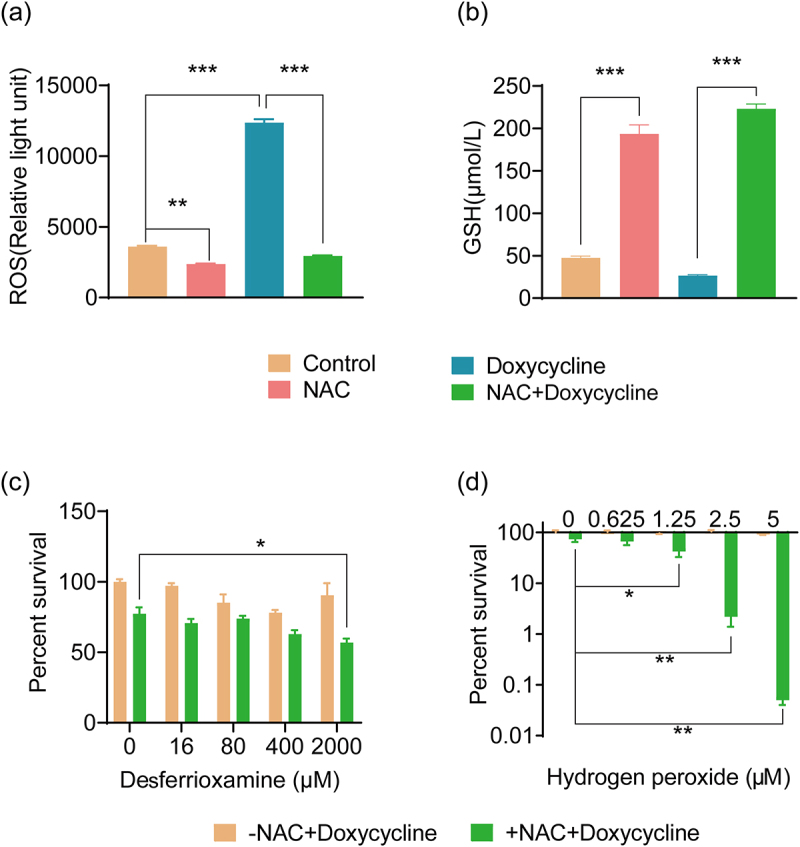
ROS affects the antibacterial efficacy of doxycycline hydrochloride. (a) Effect of NAC on ROS content of ATCC15947 by measuring the relative light unit of DCFH-DA. (b) Effect of NAC on GSH content in ATCC15947. (c-d) effects of exogenous desferrioxamine or hydrogen peroxide on NAC promoting doxycycline resistance. NAC: 40 mM; doxycycline: 40 μg/ml. Data are presented as mean ± SEM (*n* = 3 biological replicates) and statistically significant differences are identified by t-test. *p < 0.05.**p < 0.01. ***p < 0.001.

Metabolites of the NAC-activated amino acid pathways, such as cysteine and methionine, are involved in glutathione (GSH) synthesis. Among the upregulated DEPs, five were related to GSH synthesis (Table S3). Therefore, we speculated that NAC may act as an antioxidant by activating the GSH pathway. First, the ROS levels were measured (Figure 4a). Compared with the control group, the intracellular ROS levels of bacteria in the NAC-treated group were reduced. Similarly, compared with the doxycycline group, the bacterial intracellular ROS levels in the NAC+Doxycycline group were also significantly reduced. Thus, NAC reduced intracellular ROS levels. Next, the GSH content was measured. Compared with the control group, the GSH content in the bacterial cells increased significantly after NAC treatment. Similarly, compared with the doxycycline group, the intracellular GSH content of bacteria in the NAC+Doxycycline group also increased significantly (Figure 4b). Adding high concentrations (2 mM) of the GSH inhibitor desferrioxamine increased the sensitivity of *E. tarda* to doxycycline (Figure 4c). ROS levels are crucial for antibiotic lethality and hydrogen peroxide can increase ROS levels. Hydrogen peroxide treatment increased *E. tarda* sensitivity to doxycycline in a dose-dependent manner (Figure 4d). These results suggest that NAC increases intracellular GSH content in bacteria and reduces intracellular ROS levels to protect the bacteria.

### NAC reduces antibiotic uptake and increases efflux activity

**Figure 5.**
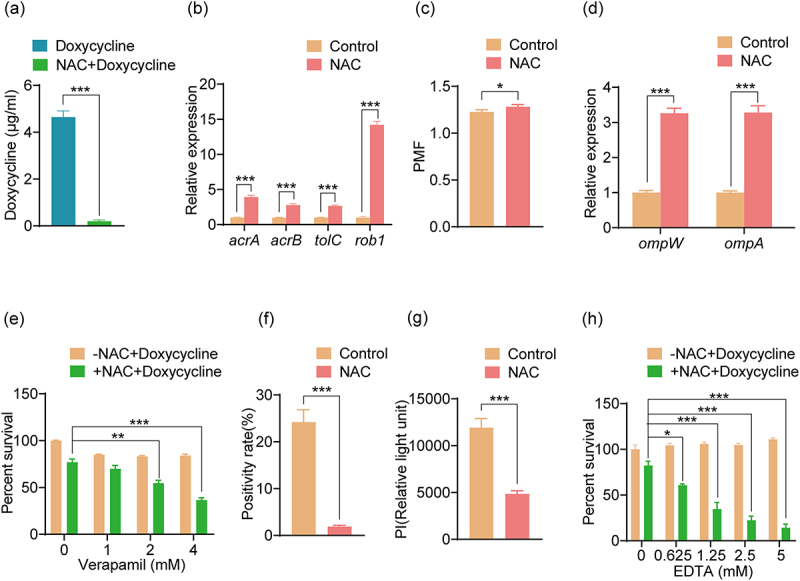
Intracellular antibiotic content affects the antibacterial efficacy of doxycycline hydrochloride. (a) The intracellular doxycycline content of ATCC15947 was monitored by liquid chromatography. (b) Effect of NAC on the expression level of efflux pump protein gene of ATCC15947. (c) The PMF of ATCC15947 was detected using DiOC_2_(3) dye. (d) Effect of NAC on the expression level of outer membrane protein gene expression level of ATCC15947. (e) Effect of exogenous verapamil on NAC promoting doxycycline resistance. (f, g) SYBR green I or PI detects the effect of NAC stimulation on ATCC15947 membrane permeability after 6 h. (h) Effect of exogenous EDTA on NAC promoting doxycycline resistance. NAC: 40 mM; doxycycline: 40 μg/ml. Data are presented as mean ± SEM (*n* = 3 biological replicates) and statistically significant differences are identified by t-test. *p < 0.05. **p < 0.01. ***p < 0.001.

The intracellular antibiotic content of bacteria plays a crucial role in their activity [37,38]. Compared with the doxycycline group, the intracellular doxycycline content in the NAC+Doxycycline group was significantly reduced (Figure 5a). There are various efflux pump systems in bacteria that pump out antibiotics to increase survival. Compared with the control group, the expression of common efflux pump genes (*acrA*, *acrB*, *tolC*, and *rob1*) was increased in the NAC group (Figure 5b), suggesting that NAC promotes the enhancement of efflux pump activity. Proton motive force (PMF) consists of the electric potential difference and the proton concentration difference across the cytoplasmic membrane. The activity of efflux pumps depends largely on PMF [10] and PMF levels were significantly increased in the NAC group compared with the control group (Figure 5c). Additionally, compared with the control group, the expression of *ompW* and *ompA*, which encode outer membrane proteins (OmpW and OmpA) that increase the activity of efflux pumps, increased in the NAC group (Figure 5d). Verapamil, an efflux pump inhibitor [39], was used to test the effect of efflux pumps. Compared with the NAC+Doxycycline group, verapamil increased the bacterial sensitivity to doxycycline (Figure 5e). In addition, we explored the impact of NAC on cell membrane permeability. Compared with the control group, the positive rate of fluorescent dye staining in the NAC-treated group was significantly lower (Figure 5f); the absorbance of the NAC-treated group was also significantly reduced (Figure 5g), suggesting that NAC reduces membrane permeability. Ethylenediaminetetraacetic acid (EDTA) disrupts cell membrane permeability [40]. Compared with the NAC+Doxycycline group, EDTA treatment increased the sensitivity to doxycycline in a dose-dependent manner (Figure 5h). These results indicate that NAC reduces antibiotic uptake via reduced membrane permeability and increased efflux.

### NAC increases protein aggregation

**Figure 6.**
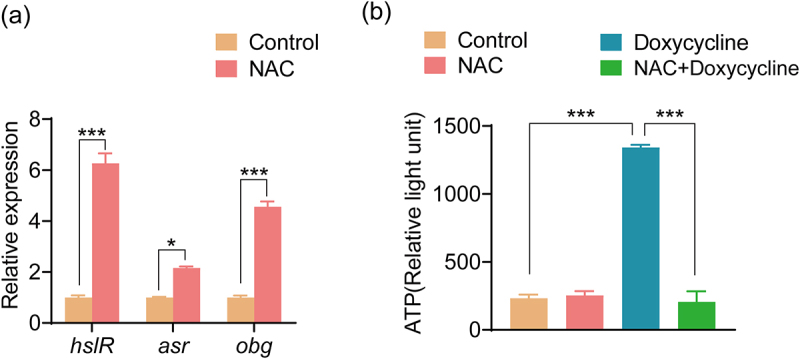
Protein aggregation affects the antibacterial efficacy of doxycycline hydrochloride. (a) Effect of NAC on the expression level of protein aggregation-related gene expression level of ATCC15947. (b) Effect of NAC on ATP content of ATCC15947 by measuring the relative light unit. NAC: 40 mM; doxycycline: 40 μg/ml. Data are presented as mean ± SEM (*n* = 3 biological replicates) and statistically significant differences are identified by t-test. *p < 0.05. ***p < 0.001.

Bacterial resistance may involve protein aggregation. Because cysteine is involved in the synthesis of many proteins and NAC is an acetyl derivative of cysteine, we hypothesized that NAC affects intracellular protein aggregation. To test this hypothesis, the expression levels of the three main genes involved in protein aggregation (*hslR, asr*, and *obgE*) [41–43] were measured. Compared with the control group, the expression levels of *hslR*, *asr*, and *obgE* in the NAC group were increased (Figure 6a). Furthermore, the insoluble protein bands in the NAC group were also increased (Figure S5). Decreased ATP levels have been reported to be associated with protein aggregation [44]. The ATP level of the NAC+Doxycycline group was significantly reduced compared with the doxycycline-alone group (Figure 6b). These results suggest that NAC protects bacteria from doxycycline by increasing protein aggregation.

## Discussion

Bacterial drug resistance is a global health crisis [45]. Drug combinations (such as non-antibiotic substances and antibiotics) are used to alleviate the harm caused by bacterial resistance [46–48]. Therefore, the exploration of non-antibiotic substances is crucial. NAC is often used in combination with antibiotics to treat infections [49,50]. Modulation of susceptibility to antibiotics by NAC has been shown in both Gram-negative and Gram-positive bacteria depending on the antibiotics used [32,51]. In contrast to previous studies, this study found that NAC promoted doxycycline resistance of *E. tarda* ATCC15947. Clinically, the combination of NAC and antibiotics may induce bacteria to become resistant. Therefore, tetracycline antibiotics and NAC are indicated to not be administered simultaneously [52,53].

We also explored the mechanism by which NAC promotes doxycycline resistance in bacteria, which provides a theoretical basis for clinical drug administration contraindications. Proteomics is an effective method applied in the field of bacterial resistance [54–56]. Our proteomics results show that NAC may regulate amino acid synthesis and metabolism pathways. In particular, many DEPs are involved in the synthesis of GSH, suggesting that NAC is related to oxidative stress.

The bactericidal activity of antibiotics is related to oxidative stress [57–59]. In *E. tarda* ATCC15947, modulation of antibiotic activity by non-antibiotic substances is closely correlation with bacterial intracellular redox levels [23,24]. Consistent with previous studies, NAC-induced resistance to doxycycline was related to low levels of intracellular ROS in bacteria. The synergistic potential of amino acids and antibiotics varies among different bacterial species [60]. Our data showed that amino acids such as cysteine increase antibiotic resistance. However, amino acids and antibiotics may also synergistically kill bacteria [61,62]. We speculated that this is due to antibiotic and bacterial species specificity. Our research suggested that NAC maintains low levels of intracellular ROS by activating the amino acid metabolism and synthesis pathway to increase GSH levels, thus inhibiting ROS production. NAC has antioxidant properties and acts as a ROS eliminator [63,64], and can reduce intracellular ROS levels through the GSH pathway [65]. This study reiterates the important role of NAC as an antioxidant in bacteria.

In addition to NAC’s antioxidant role, we also explored its other roles in bacteria. We find that NAC affects bacterial efflux pumps, outer membrane protein activity, and membrane permeability. It is worth noting that NAC can promote the antibiotic efflux. On the one hand, it activates efflux pump activity by increasing PMF levels; on the other hand, it activates outer membrane protein activity. PMF is of great significance in controlling the entry and exit of substances [66] and PMF differentially regulates antibiotic activity. Among aminoglycoside antibiotics, increased PMF is beneficial for the entry of antibiotics [67,68], but increased PMF also activates efflux pump activity and promotes the excretion of antibiotics [69–72]. Our data corresponded to the latter studies, which may be a potential reason why NAC induces resistance to tetracycline antibiotics such as doxycycline but promotes aminoglycoside antibiotics’ effects. The activity of antibiotics is also related to outer membrane proteins [73–76]. Our study confirmed the connection between tetracycline antibiotics and outer membrane proteins and that the increased expression of OmpA and OmpW outer membrane proteins is beneficial to the efflux of doxycycline. In addition, our data showed that NAC reduces antibiotic influx via a decrease in membrane permeability. Membrane permeability is a key factor in the development of drug resistance in Gram-negative bacteria [77–79], and non-antibiotic substances that disrupt membrane permeability may help treat bacterial resistance [80,81]. Thus, NAC protects bacteria from doxycycline by regulating intracellular activities in multiple ways to maintain intracellular doxycycline at low levels.

In addition, our data also showed that NAC is related to protein aggregation. Although protein aggregation has received increasing attention in recent years, the role of protein aggregation is controversial in the field of bacterial resistance. Increased protein aggregation is beneficial to the activity of antibiotics, thereby helping to kill bacteria [42] but increased protein aggregation is one of the potential reasons for bacterial survival [82]. Therefore, this may suggest that the regulation of bacterial resistance by protein aggregation is related to the type of antibiotics. Here, we found that NAC promotes protein aggregation, but the synergistic effects of NAC and different antibiotics vary. This may explain the controversy between protein aggregation and bacterial resistance and suggests that protein aggregation has antibiotic-specific effects. ATP levels affect bacterial sensitivity to antibiotics [83] and low levels of ATP promote protein aggregation [44]. Our study suggested that bacterial resistance may occur by increasing intracellular protein aggregation and maintaining lower intracellular ATP levels. Further research is required to clarify the relationship between protein aggregation, ATP, and bacterial resistance.

**Figure 7.**
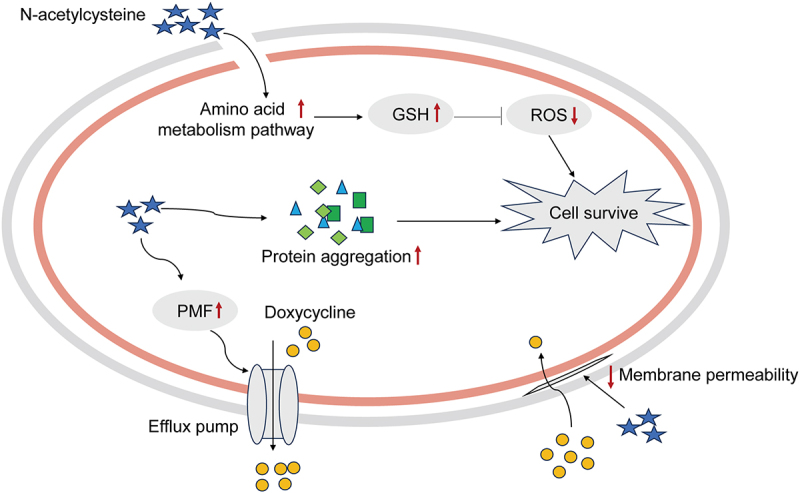
NAC combination antibiotic to restore drug resistance mechanism. NAC reduces intracellular reactive oxygen species (ROS) to offset the damage caused by oxidative stress. At the same time, NAC increases efflux pump activity and reduces membrane permeability, thereby affecting the accumulation of intracellular antibiotic content. In addition, NAC increases protein aggregation levels to resist doxycycline attack. The superposition of multiple mechanisms enables NAC to restore the resistance of ATCC15947 to doxycycline.

In summary, we explored the mechanism by which NAC promotes doxycycline resistance in *E. tarda* (Figure 7). Our research confirms the role of NAC as an antioxidant in bacteria and we show that NAC is related to efflux pump activity, outer membrane proteins, membrane permeability, and protein aggregation. Therefore, our study suggests the need to use NAC with caution as an antibiotic adjuvant and increases the understanding of the relationship between NAC and bacterial resistance.

## Materials and methods

### Bacterial strain and culture condition

The strain *E. tarda* ATCC15947 used in this study was kindly provided by Dr. Chao Wang, Shandong Freshwater Fisheries Research Institute, Jinan, China. The strain *Escherichia coli* ATCC25922 was purchased from Guangzhou Microbial Culture Collection Center. Selected a single colony on an agar plate and inoculated it into tryptic soy broth (TSB) medium. The bacteria reached saturation after 16 h of growth at 37°C and 220 rpm with shaking.

### Bacterial survival test in vitro

As found by Peng et al. [37], single colonies were picked and cultured in TSB liquid until saturated. Aspirated the bacterial liquid to collect the bacterial precipitate, and then washed it twice with 0.85% physiological saline. Resuspended the bacterial precipitate in M9 medium (10 mM acetate, 1 mM MgSO_4_, and 100 μM CaCl_2_) and adjusted the OD600 to 0.2. Then transferred to sterilized test tubes, and added 5 ml of bacterial solution to each sterilized test tube. According to the set group, added the corresponding substances into the sterilized test tube. The substances were used including NAC (Solarbio, China), doxycycline, various antibiotics, verapamil, desferrioxamine mesylate, ethylenediaminetetraacetic acid (EDTA), and hydrogen peroxide (Macklin, China). Three biological replicates were set for each group. Placed in a shaker at 37°C, 220 rpm, and incubated with shaking for 6 h. Took 100 μl of bacterial solution from the sterilized ep tube, diluted it to an appropriate multiple with 0.85% physiological saline (keep the number of colonies on the plate between 20 and 200), and pipetted 10 μl of the diluted solution onto the TSB solid plate. The number of colonies in each group was counted after overnight incubation. The bacterial time gradient sterilization experiment was to draw bacterial liquid at seven time points of incubation time of 0 h, 2 h, 4 h, 6 h, 8 h, and 10 h for spotting, and finally counted the number of colonies. Percent survival was determined by dividing the colony forming unit (CFU) obtained from a treated sample by the CFU obtained from control.

### Minimal inhibitory concentration determination

Minimum inhibitory concentrations (MIC) was tested according to the Clinical Laboratory Standards Institute (CLSI supplement M100, 2021). A single colony was picked and cultured in Mueller – Hinton broth (MHB) medium until saturated. The saturated bacteria were inoculated into fresh liquid culture medium at a ratio of 1:100 and cultured until the OD600 was 0.5. Then, the bacterial solution was diluted x100 so that the number of colonies was approximately 5 × 10^6^ CFU/ml. MHB (160 μl) was added to the first column of a 96-well plate and 90 μl MHB to each of the remaining columns. Then, 20 μl of different antibiotics was added to different wells in the first column and mixed by pipetting. 90 μl was pipetted into the next column and mixed. This operation was repeated until the end of the plate to achieve a doubling dilution effect on the antibiotics. Diluted bacterial solution (10 μl) was added to each well, so that the bacterial solution was diluted ten times, and the final number of colonies in each well was 5 × 10^5^ CFU/ml. Finally, the 96-well plate was placed in a 37°C incubator and incubated for 16 h to observe the bacterial growth status. The antibiotic concentration corresponding to the wells in which colonies were invisible to the naked eye was regarded as the MIC of the antibiotic. To ensure that the MIC value of ampicillin for *E. coli* ATCC25922 was within the range provided by CLSI, the MIC of the antibiotic was recorded.

### Protein sample preparation, LC-MS/MS analysis

Used DIA proteomics technology. Resuspended the saturated bacterial precipitate in M9 medium to an OD 600 of 1.0. Placed on a shaker at 37°C, 220 rpm, and incubated with shaking for 6 h. Collected the precipitate of 10 ml of bacterial solution for each biological replicate. The samples were then subjected to proteolysis pretreatment. Peptides were desalted using a hydrophile-lipophile balance (HLB) solid-phase extraction column (Waters, Milford, MA). The samples were then fractionated using a Shimadzu LC-15AC chromatograph and a high pH C18RP column (Bridge Peptide BEH C18, 130A 3.5um 4.6 × 250 mm, Waters). Subsequent analysed on an UltiMate 3000 high-performance liquid chromatograph and combined Q-Exactive HF mass spectrometer yielded differential proteins. Ratio = 1.2 and *p*-value = 0.05 were selected as the differential protein threshold. Finally, Skyline (version 20.2.0.343) was used to analyse the DIA data.

### Bioinformatics analysis

The software DAVID (https://david-d.ncifcrf.gov/) was used to perform enrichment analysis, and the method used was Fisher’s exact test to perform GO functional significance enrichment analysis on differential proteins. Used the KEGG database, proteins could be classified according to the pathways they participate in or the functions they perform, and KEGG pathway enrichment analysis could be performed on differential proteins grouped in pairs. The protein interaction network was done by the STRING (https://cn.string-db.org/) search tool. Samples were analysed for repeatability using Pearson correlation coefficient.

### Quantitative real-time PCR (qPCR)

We resuspended the bacterial precipitate in M9 medium to an OD 600 of 1.0. There were three biological replicates for each of the blank and NAC groups. Samples were placed in a shaker at 37°C, 220 rpm, and incubated with shaking for 6 h. A pellet of 2 ml bacterial solution was collected per sample. RNA extraction was performed using the chloroform-isoamyl alcohol method. Subsequently, genomic DNA was removed and the RNA was reverse transcribed into DNA using the Evo M-MLV reverse transcription premix kit (Accurate Biotechnology Co., Ltd., Guangdong, China). Briefly, the gDNA clean system was 10 μl, including 2 μl 5× gDNA-clean kit, RNA sample, and ddH_2_O. The reaction parameters were 42°C for 2 min. Next, reverse transcription was performed. The reaction volume was 20 μl, including 4 μl 5× Evo M-MLV RT Reaction Mix * 1, 10 μl sample, and 6 μl ddH_2_O.The reaction parameters were 37°C for 15 min followed by 85°C for 15 s. Finally, SYBR Green Pro Taq HS premixed qPCR kit (Accurate Biotechnology Co., Ltd., Guangdong, China) was used for qPCR. The entire reaction volume was 10 μl (0.5 μl upstream and downstream primers, 5 μl 2× SYBR Green Pro Taq HS Premix*, and 4 μl sample). The primer sequences information were listed in Table S1. The reaction parameters were as follows: pre-denaturation 95°C, 30 s; cycle reaction: 95°C, 5 s; 58°C, 30 s, 45 cycles. The results were analysed using the threshold cycle (Ct) value to obtain the relative expression of the target gene and the internal reference gene (16S rRNA). Data was calculated by the 2^−ΔΔCt^ method.

### Insoluble protein isolation and SDS-PAGE

Insoluble protein isolation was performed as previously described with a few modifications [84]. The overnight cultures were adjusted to OD 600 = 1.0 in M9 medium. With or without 40 mM NAC was used to treat bacteria and placed in a shaker at 37°C, 220 rpm, and incubated with shaking for 6 h. The precipitate of 10 ml of bacterial solution was collected for each sample. The precipitate was suspended in 40 μl of buffer A (10 mM potassium phosphate buffer, pH 6.5; 1 mM EDTA; 20% (w/v) sucrose; 1 mg/ml lysozyme) and incubated on ice for 30 min. Cell lysates were mixed with 360 μl of buffer B (10 mM potassium phosphate buffer, pH 6.5, 1 mM EDTA) and sonicated while cooling. The precipitated fraction was resuspended in 400 μl of buffer C (buffer B with 2% NP40) to solubilize membrane proteins and centrifuged (4°C, 15, 000 g, 30 min). The precipitate was resuspended in 400 μl Buffer B and sonicated, washed twice with 400 μl Buffer B, and resuspended in 50 μl Buffer B. Next, 12% SDS-PAGE was performed to separate the insoluble proteins in the sample. The gel was stained with 0.5% Coomassie Brilliant Blue R250, 40% methanol, 10% acetic acid, and 49.5% H_2_O for 30 min, and destained with destaining solution (70% H_2_O, 20% ethanol, and 10% acetic acid) overnight. Darker bands indicated higher levels of insoluble protein in the sample.

### Determination of reduced glutathione content

We adjusted the saturated bacteria in M9 medium to an OD600 of 1.0. 40 mM NAC and 40 μg/ml doxycycline were used to treat bacteria. Samples were placed in a shaker at 37°C, 220 rpm, and incubated with shaking for 6 h. The pellet from 10 ml of bacterial solution was collected for each sample, resuspended in PBS, and then sonicated for 10 min (200 W total power with 35% output, 2 s pulse, 3 s pause over ice). This was followed by centrifugation and the supernatant was collected. Glutathione (GSH) content was determined according to the instructions of the GSH determination kit (Nanjing iancheng Bioengineering Institute, China). The absorbance value of each well minus the absorbance value of the blank was the final absorbance value of each well. The standard curve was based on the absorbance values corresponding to GSH standard solutions of different concentrations. Finally, the GSH concentrations corresponding to different samples were obtained according to the standard curve.

### Reactive oxygen species (ROS) content determination

Used 2″,7″-Dichlorodihydrofluorescein diacetate (DCFH-DA) to measure intracellular ROS content [85]. Resuspended the saturated bacterial precipitate in M9 medium to an OD 600 of 0.2. NAC and doxycycline were used to treat bacteria. Placed in a shaker at 37°C, 220 rpm, and incubated with shaking for 6 h. Took 196 μl of bacterial solution from each sample in a 96-well plate, then added 4 μl of DCFH-DA (Sigma, United States) (final concentration-20 μM), and incubated in a 37°C incubator in the dark for 1 h. Finally, a microplate reader (CLARIO Star Plus, Germany) was used for detection. Instrument parameters: excitation wavelength was 485 nm, emission wavelength was 515 nm. The absorbance value indicated the ROS content.

### Adenosine triphosphate (ATP) content determination

As found by Cheng et al. (2019) [86], BacTiter-GloTM Microbial Cell Viability Assay (Cat. G8231, Promega, Madison, WI, United States) was used to measure intracellular ATP content. Resuspended the saturated bacterial precipitate in M9 medium to an OD 600 of 0.2. NAC and doxycycline were used to treat bacteria. Placed in a shaker at 37°C, 220 rpm, and incubated with shaking for 6 h. Took 50 μl of bacterial solution from each sample in a 96-well plate, then added 50 μl of reagent and incubated in the dark for 5 min. Finally, a microplate reader was used to detect the fluorescence photometric value.

### Cell membrane permeability test

As found by Hou et al. and Nescerecka et al. [87,88], the saturated bacterial precipitate resuspended in M9 medium to an OD 600 of 0.2. With or without 40 mM NAC was used to treat bacteria. Samples were placed in a shaker at 37°C, 220rpm, and shaken for 6 h, then stained with SYBR Green I dye (Biosharp, China) at a 1:100 ratio. Samples were placed in a 37°C incubator and incubated for 30 min. Then a flow cytometer (Beckman Coulter, Brea, CA, United States) was used to measure fluorescein isothiocyanate (FITC). The threshold for forward (FSC) and side scatter (SSC) parameters was set to 8,000 and for number of bacteria was set to 10,000. The proportion of fluorescence to bacterial migration was the positive rate of staining. Cells were stained with propidium iodide (PI) dye (Yeasen, China) at a ratio of 1:100 to a final dye concentration of 0.1 μg/ml. After incubation in the dark for 30 min, 200 μl of each group was placed in a 96-well plate, and the absorbance value was measured using a microplate reader (excitation: 535 nm, emission: 615 nm). The absorbance value indicated the positive rate of bacteria stained by PI dye, which shows the permeability of the bacterial membrane.

### Proton motive force (PMF) measurement

As found by Xu et al. [89], bacterial pellets were resuspended in M9 medium to an OD 600 of 0.2. NAC was used to treat bacteria. Placed in a shaker at 37°C, 220rpm, and shook for 6 h. The removed bacterial solution was diluted 10 times and the dye 3,3′-Diethyloxacarbocyanine (DiOC_2_(3)) (Invitrogen, United States) was diluted with dimethyl sulphoxide (DMSO). Then added the dye to the bacterial solution at a ratio of 1:100 so that the final concentration of the dye was 10 μM. Incubated in the dark for 1 h, and finally detected using flow cytometry. The value was calculated from the formula. The numerical value calculated experimentally indicated the level of PMF.

$$1.5+\log10\left(\frac{\mathrm{red}\;\mathrm{fluorescence}}{\mathrm{green}\;\mathrm{fluorescence}}\right)$$

### Determination of bacterial intracellular doxycycline concentration

The instrument used was an analytical liquid chromatograph (Agilent, USA). First, a doxycycline standard curve was developed. Five doxycycline standard solutions were prepared with different concentrations and filtered with a 0.22-μm filter. As in Hadad et al. [90], the mobile phase was a mixture of 20 mM potassium dihydrogen phosphate and acetonitrile. The instrument parameters were as follows: flow rate 1.5 ml/min, injection volume 20 μl, and detection wavelength 245 nm. Through the obtained peak area values, a doxycycline standard curve was drawn. Next, the bacterial pellet was resuspended in M9 medium to an OD 600 of 0.2, placed in a shaker at 37°C, 220 rpm, and incubated with shaking for 6 h. The doxycycline group and the NAC+Doxycycline group were treated with corresponding substances (40 mM NAC, 40 μg/ml doxycycline) respectively, and each group had three biological replicates. The bacterial pellet from 100 ml of cultures was collected for each sample and resuspended in 20 mM potassium dihydrogen phosphate, sonicated for 10 min (200 W total power with 35% output, 2 s pulse, 3 s pause over ice), and centrifuged. The supernatants were filtered using a 0.22-μm filter. The doxycycline content in the sample was determined by comparison with the standard curve.

### Scanning electron microscope (SEM) analysis

Adjusted the saturated bacteria in M9 medium to an OD 600 of 0.2. 40 mM NAC and 40 μg/ml doxycycline were used to treat bacteria. Placed in a shaker at 37°C, 220 rpm, and incubated with shaking for 6 h. Collected the precipitate of 10 ml of bacterial solution for each sample. Then resuspended the precipitate in electron microscope fixative and fixed at room temperature for 2 h. The fixed samples were rinsed three times with 0.1 M phosphate buffer (pH 7.4) for 15 min each time. Prepared 1% osmic acid in 0.1 M phosphate buffer (pH 7.4) and fixed at room temperature in the dark for 1–2 h. Rinsed 3 times with 0.1 M phosphate buffer (pH 7.4), 15 min each time. Then used alcohol for gradient dehydration and drying. Finally, the sample was conductively processed, and the sample was placed tightly on the conductive carbon film double-sided tape and placed on the sample stage of the ion sputtering instrument for about 30 seconds. Observed and collected pictures under a scanning electron microscope (Hitachi, SU8100, Japan).

## Supplementary Material

Supplementaryfigure1.tif

Supplementary Figure 2.tif

Supplementary Figure 5.tif

Supplementary Figure 4.tif

Supplementary Figure 3.tif

## Data Availability

The data generated during the study is available at repository name “Proteomic data of ATCC15947 without or with NAC” at https://doi.org/10.6084/m9.figshare.25908706
