# Supplementary figures and images for: N-acetylcysteine promotes doxycycline resistance in the bacterial pathogen Edwardsiella tarda
Source: Virulence. 2024 Sep 6;15(1):2399983. doi: 10.1080/21505594.2024.2399983 (PMC11409502; doi:10.1080/21505594.2024.2399983)

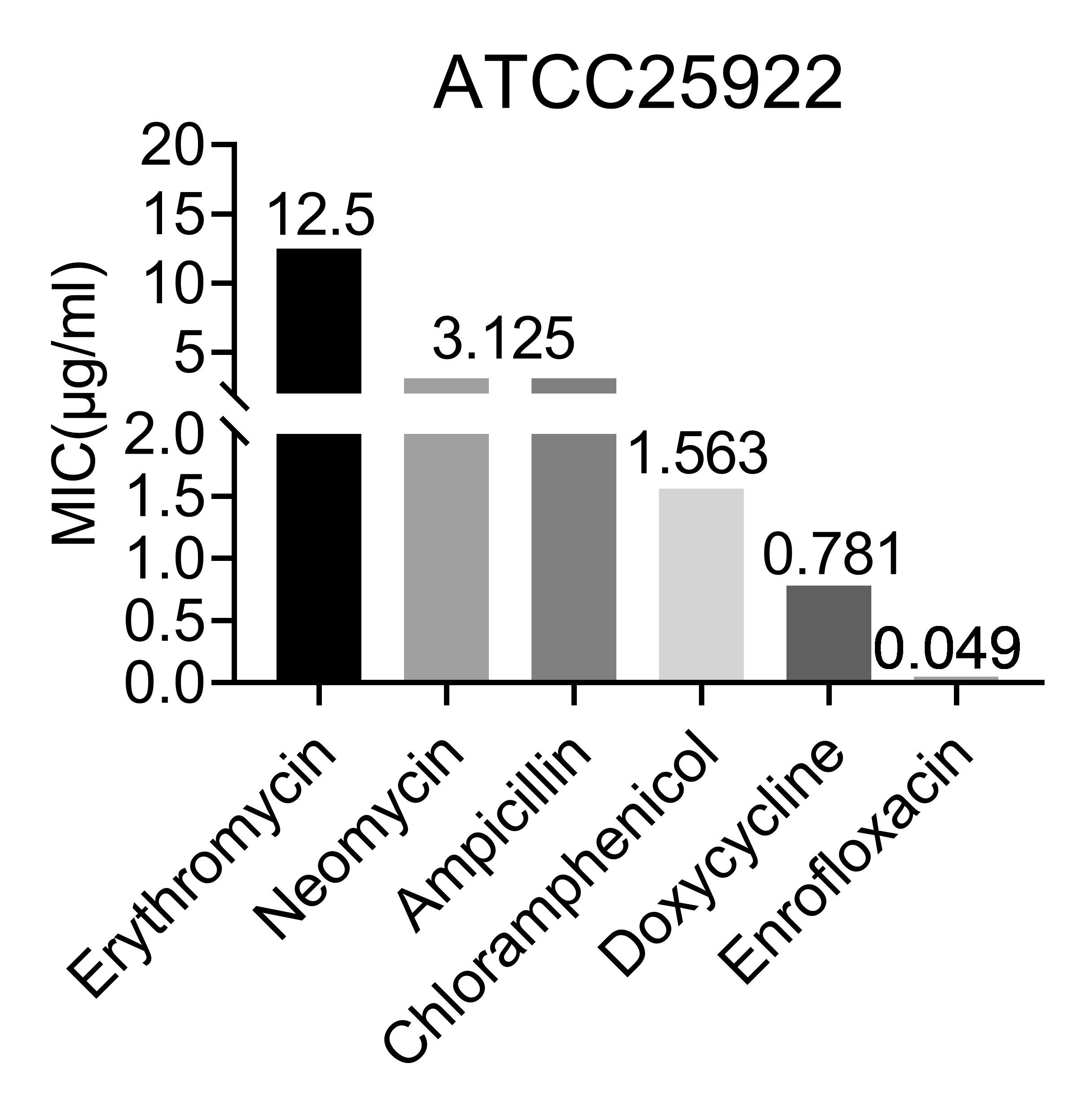

Supplement: Supplementaryfigure1.tif [file KVIR_A_2399983_SM9698.tif]

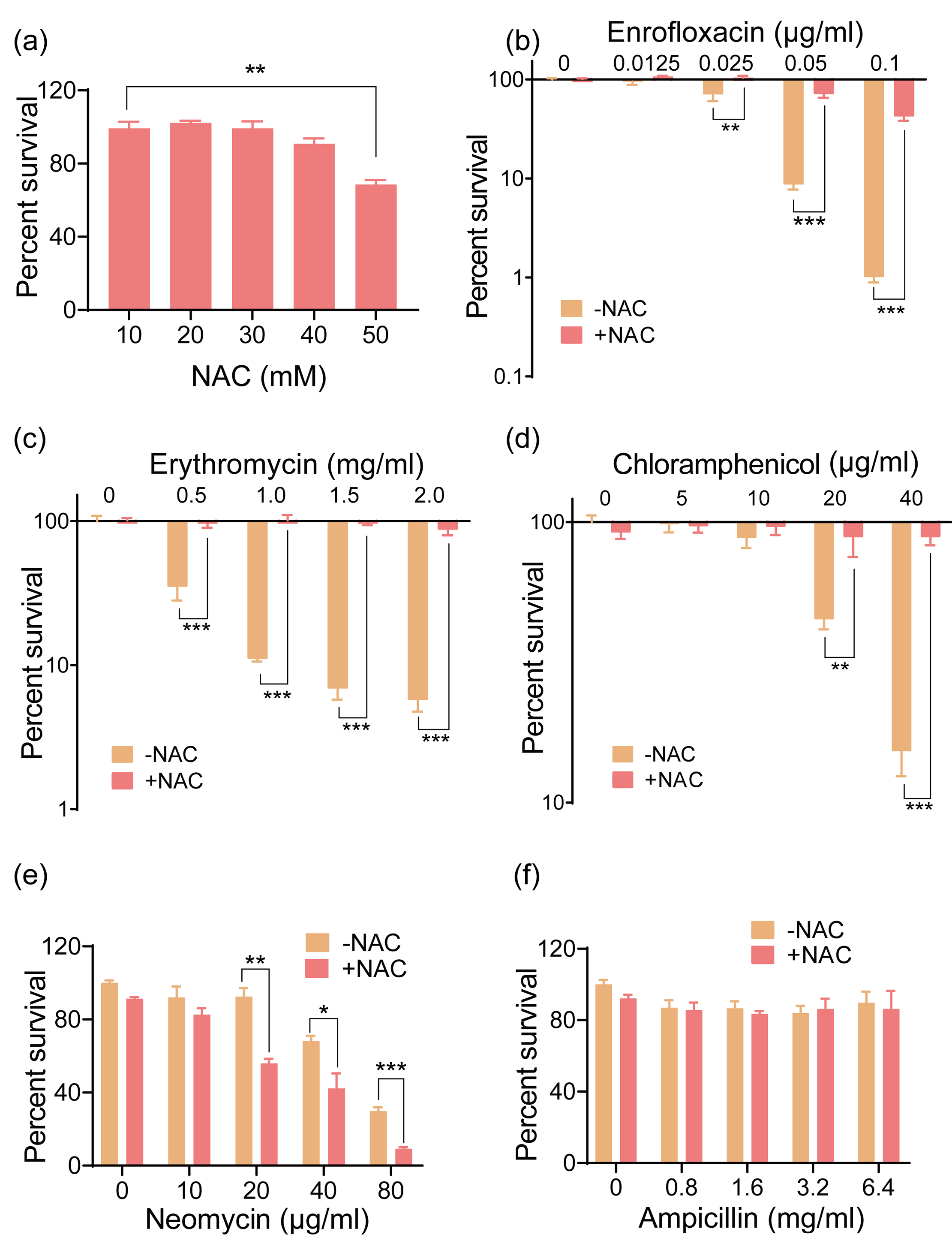

Supplement: Supplementary Figure 2.tif [file KVIR_A_2399983_SM9697.tif]

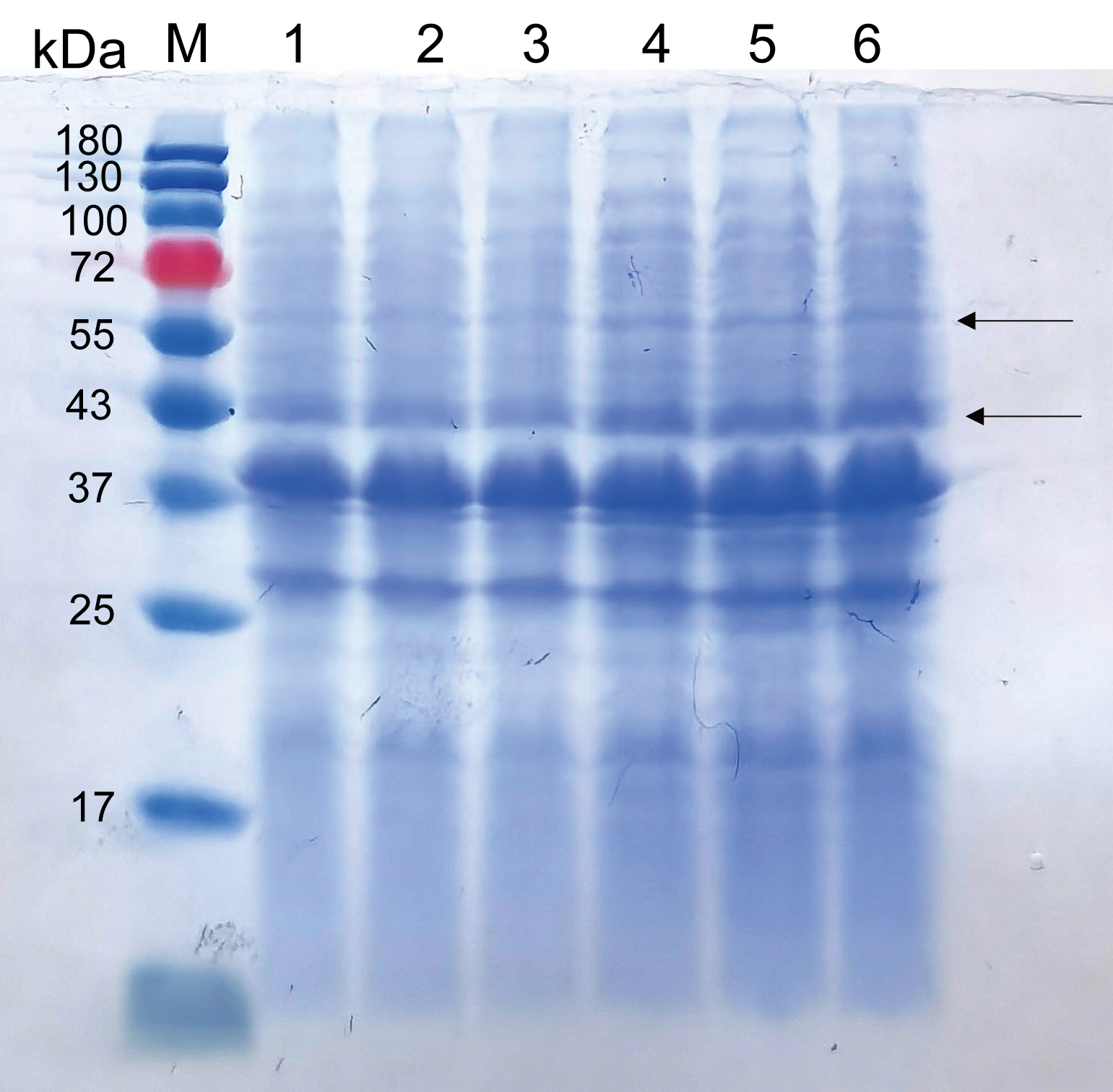

Supplement: Supplementary Figure 5.tif [file KVIR_A_2399983_SM9696.tif]

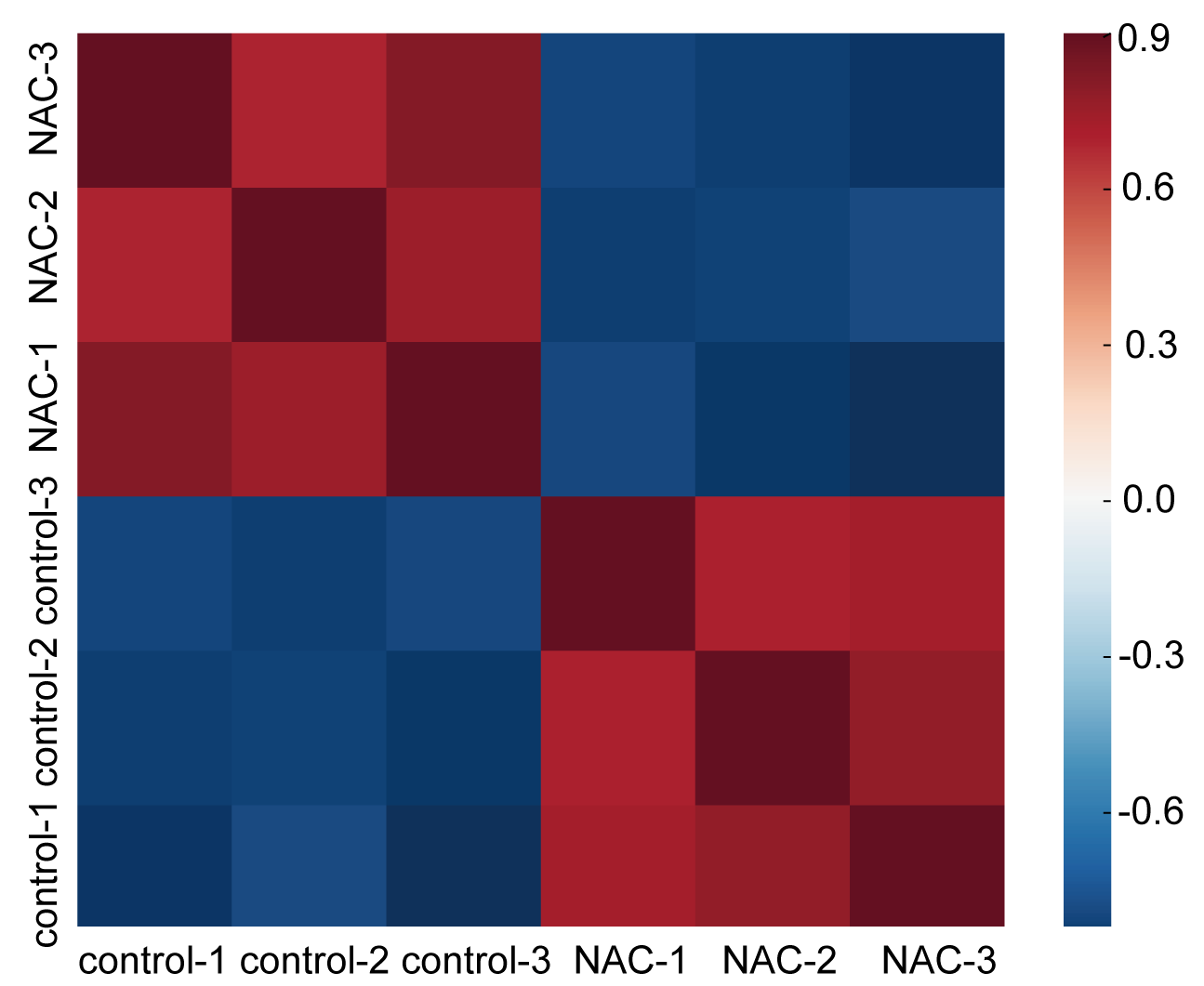

Supplement: Supplementary Figure 4.tif [file KVIR_A_2399983_SM9695.tif]

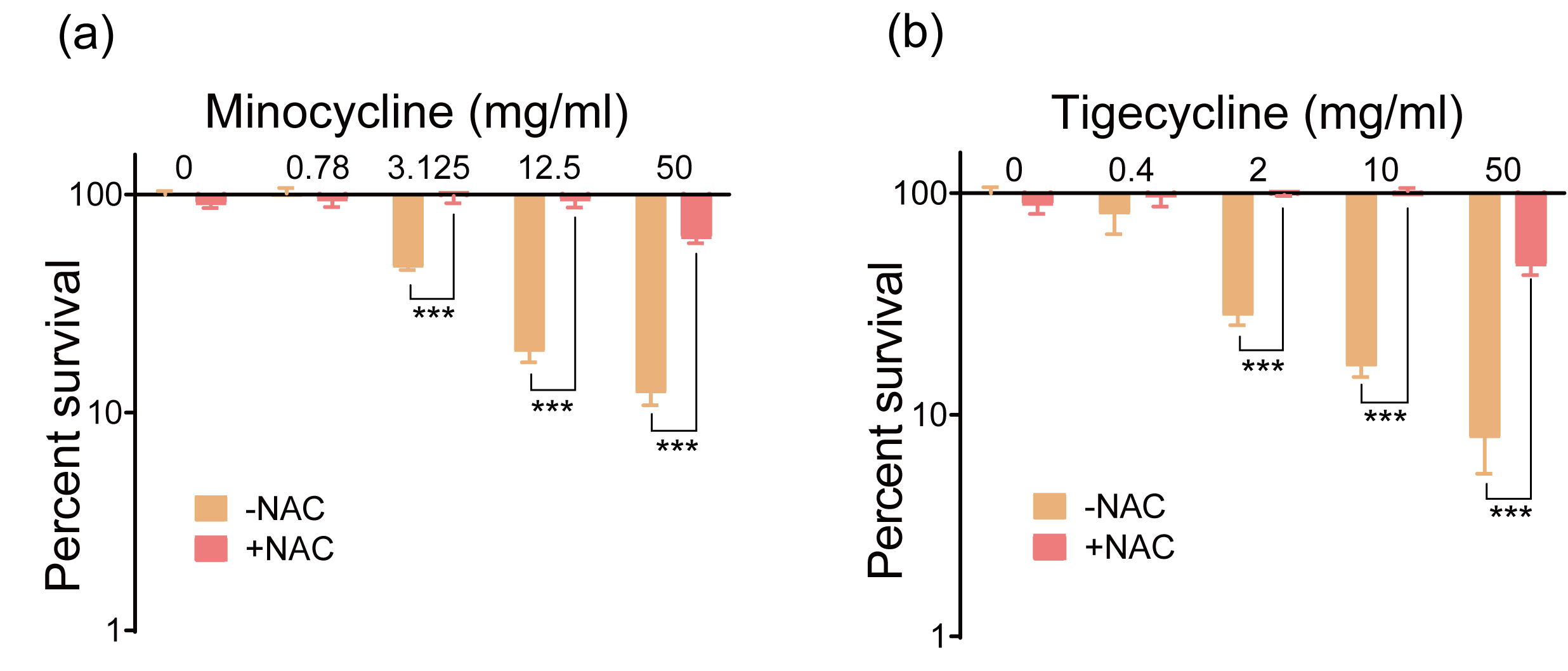

Supplement: Supplementary Figure 3.tif [file KVIR_A_2399983_SM9694.tif]
